# Distribution in the brain and possible neuroprotective effects of intranasally delivered multi-walled carbon nanotubes†

**DOI:** 10.1039/d0na00869a

**Published:** 2020-11-12

**Authors:** Marzia Soligo, Fausto Maria Felsani, Tatiana Da Ros, Susanna Bosi, Elena Pellizzoni, Stefano Bruni, Jacopo Isopi, Massimo Marcaccio, Luigi Manni, Silvana Fiorito

**Affiliations:** Institute of Translational Pharmacology, CNR Via Fosso del Cavaliere 100 00133 Rome Italy silvana.fiorito@ift.cnr.it; INSTM – Trieste Unit, Department of Chemical and Pharmaceutical Sciences, University of Trieste Via Licio Giorgieri 1 34127 Trieste Italy; Former Medical Director Sanofi - Genzyme, Italy, currently Orchard Therapeutics 108 Cannon Street London UK; Department of Chemistry “G. Ciamician”, University of Bologna Via Selmi, 2 40126 Bologna Italy

## Abstract

Carbon nanotubes (CNTs) are currently under active investigation for their use in several biomedical applications, especially in neurological diseases and nervous system injury due to their electrochemical properties. Nowadays, no CNT-based therapeutic products for internal use appear to be close to the market, due to the still limited knowledge on their fate after delivery to living organisms and, in particular, on their toxicological profile. The purpose of the present work was to address the distribution in the brain parenchyma of two intranasally delivered MWCNTs (MWCNTs 1 and a-MWCNTs 2), different from each other, the first being non electroconductive while the second results in being electroconductive. After intranasal delivery, the presence of CNTs was investigated in several brain areas, discriminating the specific cell types involved in the CNT uptake. We also aimed to verify the neuroprotective potential of the two types of CNTs, delivering them in rats affected by early diabetic encephalopathy and analysing the modulation of nerve growth factor metabolism and the effects of CNTs on the neuronal and glial phenotypes. Our findings showed that both CNT types, when intranasally delivered, reached numerous brain areas and, in particular, the limbic area that plays a crucial role in the development and progression of major neurodegenerative diseases. Furthermore, we demonstrated that electroconductive MWCNTs were able to exert neuroprotective effects through the modulation of a key neurotrophic factor and probably the improvement of neurodegeneration-related gliosis.

## Abstract 2

Carbon nanotubes (CNTs) are currently under active investigation for their use in several biomedical applications, especially in neurological diseases and nervous system injury due to their electrochemical properties.

## Graphical abstract

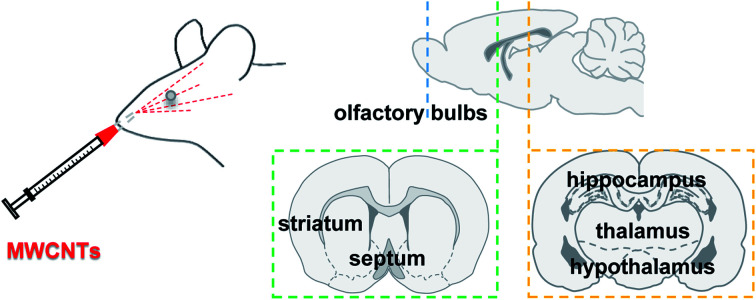

## 1 Introduction

Carbon nanotubes (CNTs), due to their unusual and unique combination of mechanical, electrical, chemical and physical properties, are under active investigation for their use in several biomedical applications. Their potential exploitation for therapeutic purposes in neurological diseases and nervous system injury is currently the most promising one from a clinical and experimental point of view.^1–3^ In the last few years CNTs were investigated for their beneficial use in nervous system tissue engineering and recent research increasingly focused on their exploitation as nanotools to modulate neurobiological processes, such as growth and organization of neural networks, in order to accomplish nerve tissue reconstruction and repair.^4,5^ CNTs were shown to be promising nanomaterials for new tissue-engineered products such as prosthetic neuronal devices^6–8^ as well as drug delivery devices^9^, to affect neurons, astrocytes and microglia cell functions^10–12^ and to sustain and promote electrical activity in networks of cultured neurons.^13^ Moreover, functionalized carbon nanotubes (f-CNTs) were reported to induce neurite outgrowth^14,15^ and CNT substrates are known to boost neuronal electrical signalling increasing GABAergic and glutamatergic synaptogenesis^16,17^ and to directly stimulate brain circuit activity.^13^ Moreover, electroconductive multi-walled carbon nanotube (MWCNT)-incorporated scaffolds were demonstrated to promote neural stem cell proliferation and early neuronal differentiation.^18^

It is currently clear that the electrochemical properties of CNTs underlie their effects on the nerve tissue system, nevertheless the mechanisms governing their interactions with these cells at the ultrastructural level have been little explored. Therefore, it is very important to investigate the mutual interaction between these nanomaterials and the neuronal cell system, in consideration of the peculiar sensitivity of these cells to electrical stimuli.

Although CNTs serve as an important niche in theranostic (therapeutic and diagnostic) nanomedicine,^19^ no CNT-based therapeutic product for internal use appears to be close to the market. Some major obstacles to their clinical development are related to their fate after delivery to living cells and organisms, which needs to be suitably studied and determined together with their toxicological profile. Implemented knowledge on the *in vivo* biodistribution of CNTs would therefore provide the basis for further development of targeted CNT delivery systems to specific tissues for diagnostic and therapeutic uses. CNT intravenous administration seems to be of special value for the characterization of their accumulation profiles in tissues, but it seems disadvantageous for the selective delivery of CNTs to the brain parenchyma, since it leads to a widespread distribution into many organs. Moreover, high systemic doses are needed to achieve therapeutic levels in the brain parenchyma, which can lead to adverse effects in the body. There are still few studies on the distribution and fate of these nanomaterials following their direct administration to the neuronal tissue.^1,20–24^ The potential of the intracranial injection route to assess the brain distribution and fate of CNTs following their stereotactic administration to the cerebral cortex has been investigated. Nevertheless, the intracranial delivery of drugs and/or nanomaterials, though of primary clinical utility, is limited by the invasiveness of the technique and patient compliance. The intranasal route has recently gained attention, since it allows effective delivery of drugs to the brain tissue by the olfactory and trigeminal nerve.^25^ Besides that, intranasal drug delivery is a non-invasive method that rapidly targets therapeutics to the Central Nervous System (CNS), bypassing the blood-brain barrier (BBB) and providing decreased systemic exposure of the drug and limited degradation of therapeutics.^26^

In order to explore the feasibility of their administration *via* the nasal route, we addressed whether CNT intranasal delivery could be effective for reaching all areas of the brain. Besides assessing their distribution in the brain parenchyma and their uptake by selected cell populations in the brain, we aimed to verify the neuroprotective potential of electro-conductive CNTs. For this purpose we first performed a preliminary characterization of the displacement in the brain of both pristine and annealed electroconductive MWCNTs delivered *via* the olfactory route; then we investigated *in vivo* their neuroprotective potential, by delivering them to rats affected by early diabetic encephalopathy, characterized by mild neurodegeneration and poor neurocognitive performances.^27–29^

## 2 Experimental methods

### 2.1. Carbon nanotubes

Pristine multi-walled carbon nanotubes (MWCNTs-P) (CRMD, Orléans, France) were synthesized, as previously described,^30^ by a regular catalyst-assisted chemical vapour deposition technique (CCVD) during which a gaseous hydrocarbon (acetylene) is cracked at 600 °C in the presence of CoMgO solid solution as a catalyst.^31^ Following preparation, exposed catalyst particles were dissolved in 12 mol L^−1^ HCl solution. The MWCNTs obtained were in part used as-prepared (MWCNTs), and in part annealed at 2400 °C under an argon atmosphere (a-MWCNTs). Briefly, the annealing took place in a high temperature furnace equipped with a vitreous carbon resistor, under argon flow (3 L min^−1^). The heating rate was 20 °C min^−1^ and the residence time at the determined temperature was 15 min (ref. 32). The outer diameters of the produced tubes were in the 10–20 nm range^32^ and the average number of walls was ∼15. The degree of purity and the morphology, structure, and nanotexture of both MWCNT samples were evaluated by X-ray diffraction (XRD), X-ray photoelectron spectroscopy (XPS) and transmission electron microscopy (TEM) and correspond to what was previously reported^30^ confirming one more time that the annealing treatment eliminated any trace of catalysts and reduced the content of oxygen, while maintaining their integrity, their structure, and their shape.

### 2.2. Carbon nanotube functionalization

**Fig. 1:**
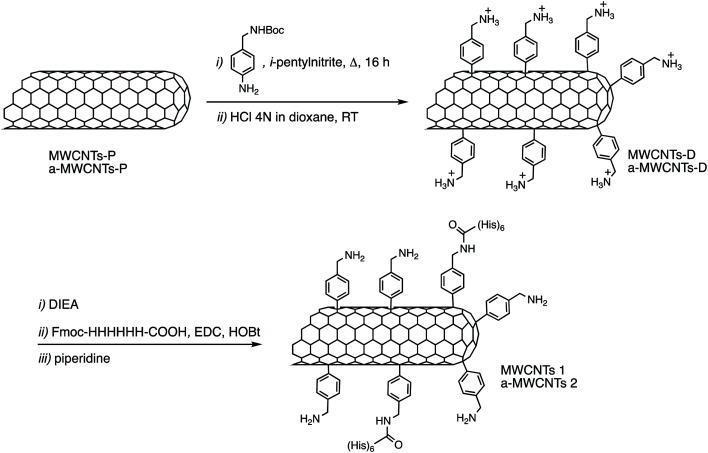
Synthesis of derivatives MWCNTs 1 and a-MWCNTs 2.

12 mg of MWCNTs-P or a-MWCNTs-P were suspended in 10 mL of MilliQ water and sonicated for 30 min. Then 100 mg (0.4 mmol) of 4-(*N*-Boc-aminoethyl) aniline and 100 μL (43.6 mg, 0.8 mmol) of isopentyl nitrite were added and the mixture was stirred at 80 °C for 16 h (Fig. 1). The black solid was left to settle and the yellow supernatant was discarded by filtration using a hydrophilic JHWP Omnipore™ filter (0.44 μm) and the tubes were washed with DMF, MeOH, and water and directly suspended in 6 mL of HCl 4 N in dioxane and stirred at room temperature (RT), overnight. The functionalization of the tubes by the Tour reaction^33^ allowed to obtain MWCNTs-D and a-MWCNTs-D, respectively from MWCNTs-P and a-MWCNTs-P. The products were filtered using a hydrophilic JHWP Omnipore™ filter (0.1 μm) and washed with MilliQ water, MeOH, DCM, and diethyl ether. 8.5 mg of MWCNTs-D and 8.3 mg of a-MWCNTs-D were obtained. 5 mg of MWCNTs-D or a-MWCNTs-D were dispersed in 1 mL of DMF and sonicated for 15 min. Then DIEA (2.2 mg, 3 μL) was added and the mixture was stirred for 30 min. At the same time 2.6 mg of Fmoc-His_6_-COOH (2.4 μmol) were dissolved in 1 mL of DMF together with 0.5 mg of EDC HCl and 0.33 mg of HOBt. The solution was stirred for 30 min and then the two preparations were mixed and the His-tag was incubated over night. After filtration and washing, the MWCNT derivatives were dispersed in 2 mL of DMF and directly treated with 400 μL of piperidine. The mixture was stirred overnight and then the product was filtered and washed with DMF, MeOH, DCM, and diethyl ether, obtaining 4.3 mg and 4.8 mg of MWCNTs 1 and a-MWCNTs 2, respectively (Fig. 1). Pristine MWCNTs (MWCNTs-P and a-MWCNTs-P), functionalized MWCNTs (MWCNTs-D and a-MWCNTs-D) and His-tag conjugates (MWCNTs 1 and a-MWCNTs 2) were characterized by thermogravimetric analysis (TGA, Fig. S1A and B†) and Raman spectroscopy (Fig. S1C†). TGA was recorded using a Q500 (TA Instruments), under an inert atmosphere (N_2_). The equilibration at 100 °C for 20 min was followed by a ramp of 10 °C min^−1^ up to 800 °C. About 1 mg of compound was used for each analysis. The degree of functionalization, expressed in μmol g^−1^, is calculated as follows:
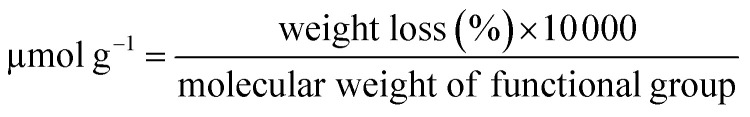
where the weight loss (%) is given by the difference between the weight loss of one compound and the weight loss of the precursor nanomaterial. The values used correspond to the weight loss that took place at 450 °C. The Raman spectra of the solid compounds were recorded using an inVia Renishaw spectrometer equipped with a 532 nm laser.

### 2.3. Electrochemical characterization

The electrochemical characterization was carried out in a glass cell equipped with a platinum spiral as a counter electrode and a saturated KCl calomel reference electrode (SCE). The CNT film was deposited as a 6 mm diameter disk, by drop-casting a 40 μL of a 1 mg mL^−1^ sonicated suspension of CNTs in PBS on an indium tin oxide (ITO) coated glass slide.^11^ The 40 μL were deposited in 4-steps of 10 μL each (confining the drop within an O-ring), followed by drying under vacuum for 45 min. Such a stepwise deposition-drying procedure allows obtaining a more homogeneous and tightly adsorbed film onto the ITO surface.^34^ The open circuit potential (OCP, Fig. S2†) and cyclic voltammetry measurements (Fig. S3†) were performed at RT in a PBS electrolyte solution by using a Biologic potentiostat SP300 instrument.

### 2.4. Assessment of MWCNT functionalization

To evaluate the correct interaction of the primary anti-His antibody with the His-tag linked to the MWCNTs, CNTs were dispersed in ultrapure water at a concentration of 100 μg mL^−1^. Ten microliters of dispersed nanotubes (both functionalized and non-functionalized) were placed in polystyrene tubes and allowed to react for 10 min with mouse anti-6xHis antibodies (Table S1†). CNT suspensions were then briefly centrifuged (500 × *g*), the supernatant was removed and the CNTs were washed 3 times with PBS, each time by suspending and briefly spinning them at 500 × *g*. CNTs were then suspended in 100 μL PBS + anti-mouse-HRP antibody (1 : 10 000, Table S1†). After a 5 min incubation, CNTs were washed with PBS as described above and suspended in 100 μL of 3,3′,5,5′-tetramethylbenzidine (TMB) liquid substrate (Thermo Scientific cat# N301). The development of the colorimetric reaction (Fig. S4†) indicates the presence of the His-tag associated with the CNTs.

### 2.5. Animals

Male Wistar rats (251–275 g) were purchased from Charles River Laboratories s.r.l. (Calco, Lecco, Italy). Rats were weighed, ear and tail tagged and housed in groups of two-three per cage with standard food and water available *ad libitum*. The animal room had a controlled 12 h light cycle (lights on at 07:00 h), lux level (on average 100 lux), temperature (21 ± 1 °C) and relative humidity (50 ± 5%). Animal care procedures were conducted in conformity with the legislation for the protection of animals used for scientific purposes provided by the relevant Italian law and European Union Directive (Italian Legislative Decree 26/2014 and 2010/63/EU) and the International Guiding Principles for Biomedical Research involving animals (Council for the International Organizations of Medical Sciences, Geneva, CH). Animals were subjected to experimental protocols approved by the Veterinary Department of the Italian Ministry of Health (permit number: 192/2015-PR). All adequate measures were taken to minimize animal pain or discomfort and all surgery was performed under mild anesthesia.

### 2.6. Diabetes induction and intranasal drug delivery

Diabetes was induced by using a single intraperitoneal injection of 65 mg kg^−1^ streptozotocin (STZ) dissolved in citrate buffer, pH 4.5 (for the experimental design see Fig. S5†). Sixty rats were randomly divided into two groups as follows: thirty rats were injected with 20 mM L^−1^ citrate, pH 4.5 (healthy groups) and 30 rats were injected with STZ as described above (diabetic groups). One week after STZ treatment, the rats were checked for the establishment of diabetes with an Accutrend™ GC (Roche Diagnostic, Germany) glucose analyzer. Rats with blood glucose levels above 300 mg dL^−1^ were allocated to the diabetic experimental groups. Before euthanasia, healthy and diabetic conscious rats were exposed to intranasal administrations of MWCNTs 1, a-MWCNTs 2 or saline once a day for one or three days. Rats received 72 μg per day of MWCNTs dispersed in solution (two applications of 9 μL for each nostril for a total of 36 μL) for each day of treatment (maximum amount of CNTs: 216 μg per rat). Rats were euthanized five weeks after STZ injection (Fig. S5†).

### 2.7. Immunofluorescence and confocal microscopy

Rats were deeply anesthetized and transcardially perfused with 4% paraformaldehyde. The brain region spanning +7.5/−5.00 mm relative to Bregma was analyzed^35^ and one section every 120 μm (1 in 3 sections), throughout the anatomic region of interest, was processed for each staining series. At least 3 sections were analyzed for each animal in every analysis performed. Free-floating coronal sections (40 μm-thick) from both hemispheres were pre-incubated with PBS containing 10% (v/v) goat serum, 1% (w/v) bovine serum albumin (BSA) and 0.5% (v/v) Triton X-100 for 1 h at RT. Sections were then incubated overnight at 4 °C with primary antibodies diluted in a PBS solution containing 1% (v/v) goat serum, 1% (w/v) bovine serum albumin (BSA) and 0.2% (v/v) Triton X-100 antibodies (Table S1†). After washing with PBS, sections were incubated 1 h at RT with specific secondary antibodies (Table S1†). After extensive washes with PBS, sections were incubated for 10 min with Hoechst reagent for nuclei staining. Stained sections were imaged with a confocal laser-scanning microscope (Leica SP5, Leica Microsystems, Wetzlar, Germany) in sequential mode, to avoid crosstalk between channels. Confocal image acquisition was conducted so that all samples were imaged using consistent settings for laser power and detector gain. Boundaries and subdivisions of the brain structures were identified with reference to the Paxinos' Rat Brain Atlas.^35^ Image processing was done by using Adobe Photoshop CS6 software: production, image brightness and contrast were enhanced by using the linear histogram correction and slightly oversaturated.

### 2.8. Enzyme-linked immunosorbent assay (ELISA)

Tissue samples from the rat brain (hippocampus) were dissected from coronal brain slices. Samples were homogenized, using a glass tissue grinder, in 50 volumes of ice-cold Hepes 1% NP40 buffer (20 mM Hepes KOH pH 7.9, 150 mM NaCl, 0.1 mM EDTA, 0.1 mM EGTA 1% (v/v) NP40) and the resulting crude homogenates were sonicated with a Sonics Vibra Cells (Sonics & Materials, Inc., Newtown, CT, USA). Total protein content was determined using the Bio-Rad DC Protein Assay Kit (Bio Rad, Hercules, CA, USA) according to the manufacturer's instruction.

Pro Nerve Growth Factor (proNGF) and mature Nerve Growth Factor (mNGF) content in tissue lysates was measured by specific ELISA, as previously described.^36^ Briefly, capture antibody was the same for both the ELISA assays (Table S1†) and was incubated overnight at RT. The unbound antibody was removed by washing the plate once with washing buffer (0.5% (v/v) Tween-20 in PBS). After blocking 1 h at RT with 1% (w/v) BSA in PBS, the plate was rinsed with washing buffer and tissue lysates or standard curves added to the wells and incubated for 2 h at RT. The microwells were then rinsed three times and incubated with detection antibodies dissolved in blocking buffer for 2 h, at RT. Following three washes to remove the unbound detection antibody, the HRP-conjugated antibody, diluted in blocking buffer, was added and incubated for 1 h at RT. To visualize antibody reactivity, the chromogenic substrate 3′,3′,5′,5′-tetramethylbenzidine (TMB, cat. T8768, Sigma-Aldrich) was used and color development was stopped by adding HCl 1 N. The colorimetric reaction was measured in the absorbance mode at 450 nm by using a Multiskan EX ELISA reader (Thermo Fisher Scientific Laboratory).

### 2.9. Statistical analysis

The GPower program (http://www.gpower.hhu.de/en.html) was used to calculate the sample size, according to the statistical methodologies used to compare means in the different experiments. Differences among the compared means ≥30% with SD ≤ 20% of the mean within groups have been considered to obtain a power of at least 80% with an alpha level of 0.05.

Statistical analyses were performed using GraphPad Prism 5 (GraphPad Software). proNGF/mNGF ELISA and confocal image analysis data were analyzed by two-way ANOVA considering diabetes induction (disease factor: healthy *vs.* diabetic groups) and the treatment with MWCNTs (treatment factor: saline, MWCNTs 1 and a-MWCNTs 2 groups) as main factors. Multiple comparisons by the Bonferroni *post hoc* test were performed when a main and/or interaction effect was detected in the ANOVA. *P* values < 0.05 were considered statistically significant. For ELISA analysis, 4 brains for each experimental group were dissected collecting the two hemispheres separately. The olfactory bulbs, thalami and hippocampi were then considered as single samples. The resulting *n* in the statistical analysis varied between 6 and 8. For image analysis of septal (rostral) hippocampal DG brain sections, three sections, one out of four cut at the cryostat, were sampled from two/three animals in each experimental group. The mean cell counts from three sections representative of a single hippocampus (2 hippocampi per rat) were then analyzed in the two-way ANOVA, resulting in *n* = 4–7 for each experimental group.

## 3. Results

### 3.1. Preparation of His-tag conjugated MWCNTs

Two kinds of MWCNTs, differing because in one case the material was annealed after synthesis (a-MWCNTs), were functionalized using the so-called Tour's reaction, consisting of the addition of aryl-radicals, derived from diazonium salts which have been produced *in situ* starting, in this case, from the 4-[(*N*-Boc)aminomethyl]aniline (Fig. 1).^37^ The Boc-protected MWCNTs were deprotected under acidic conditions, by means of hydrochloric acid and the so-obtained MWCNTs-D were allowed to react with the Fmoc-protected hexa-histidine tag, the latter being previously activated on its free carboxylic functional group through the use of coupling agents (EDC and HOBt). After filtration and washing, the obtained derivatives were deprotected under basic conditions, in order to obtain the final derivatives MWCNTs 1 and a-MWCNTs 2.

The compounds were characterized by TGA and a degree of functionalization of 575 μmol g^−1^ and 490 μmol g^−1^ were found for MWCNT-D and a-MWCNT-D, determined using the weight loss at 450 °C (Fig. S1A and B†). The Raman analyses (Fig. S1C†) show significant differences between the annealed CNTs (a-MWCNTs) and the non-annealed ones (MWCNTs), in the case of which the D band presents a very high intensity, even higher than the G band. On the contrary, in the case of the annealed one, the D band intensity is about one tenth of the G. Moreover, it was possible to observe a slight increase of the peak at 1365 cm^−1^ for a-MWCNT-D (*I*_D_/*I*_G_ 0.211) with respect to a-MWCNT-P (*I*_D_/*I*_G_ 0.161), attributable to the functionalization. The coupling reactions with the His-tag gave a degree of functionalization of 39 and 58 μmol g^−1^ for MWCNTs 1 and a-MWCNTs 2 respectively. Both His-labelled CNT derivatives were treated with UV rays for 2 h and dissolved in sterile saline to obtain a concentration of 2 mg mL^−1^. To disperse CNTs, the compounds were sonicated in an ultrasonic bath for 2 h, reaching a maximum temperature of the bath of about 50 °C temperature at which the nanotube derivatives are stable. The preparations were used immediately after sonication. The obtained dispersion presents a corpuscle in the case of the a-MWCNTs 2 while the presence of more dispersed material is evident in the case of MWCNTs 1 (see Fig. S6†).

### 3.2. Experimental diabetes in rats

An experimental study was designed to obtain a comprehensive understanding of the brain tissue distribution of both MWCNTs 1 and a-MWCNTs 2, adopting a widely accepted animal model of streptozotocin (STZ)-induced type 1 diabetes^38^ (Fig. S5†).

Rats early develop characteristic mild cognitive impairment, associated with neuronal sufferance^29^ and dysregulation in the metabolism of protein markers of neurodegeneration, as protein tau and nerve growth factor (NGF) molecular systems.^27,28^ Saline, MWCNTs 1 or a-MWCNTs 2 were intranasally delivered to healthy and diabetic rats for one or three consecutive days, and then the animals were euthanized. As expected, STZ treatment increased blood glucose levels already one week after the diabetes induction and kept it above control levels for the entire duration of the experiment. These values remained significantly high after intranasal treatment with CNTs (data not shown), suggesting that intranasal treatments with CNTs or saline did not affect the overall glucose metabolism. Accordingly, the body weight significantly decreased in diabetic rats compared to controls, remaining lower until the end of the experiment (data not shown).

### 3.3. CNT distribution in the brain parenchyma

To investigate the fate of MWCNTs after their delivery to the brain *via* the intranasal route, we focused on their tissue distribution in the brain parenchyma in healthy rats and in rats affected by early neurodegenerative conditions. After their intranasal delivery, the CNTs are conceivably dispersed into the brain parenchyma before being internalized into brain cells, where they should be detected as a dark material confined in the cytoplasm. We first verified the co-localization in cell cytoplasm of CNTs (as the dark/black material in the bright field image) and His-tag immunodetection (Fig. S7†). The observed co-localization suggests the retaining of the His-tag by CNTs after their spreading into the brain. Then, we proceeded with the analysis of CNT displacement into selected brain areas.

**Fig. 2:**
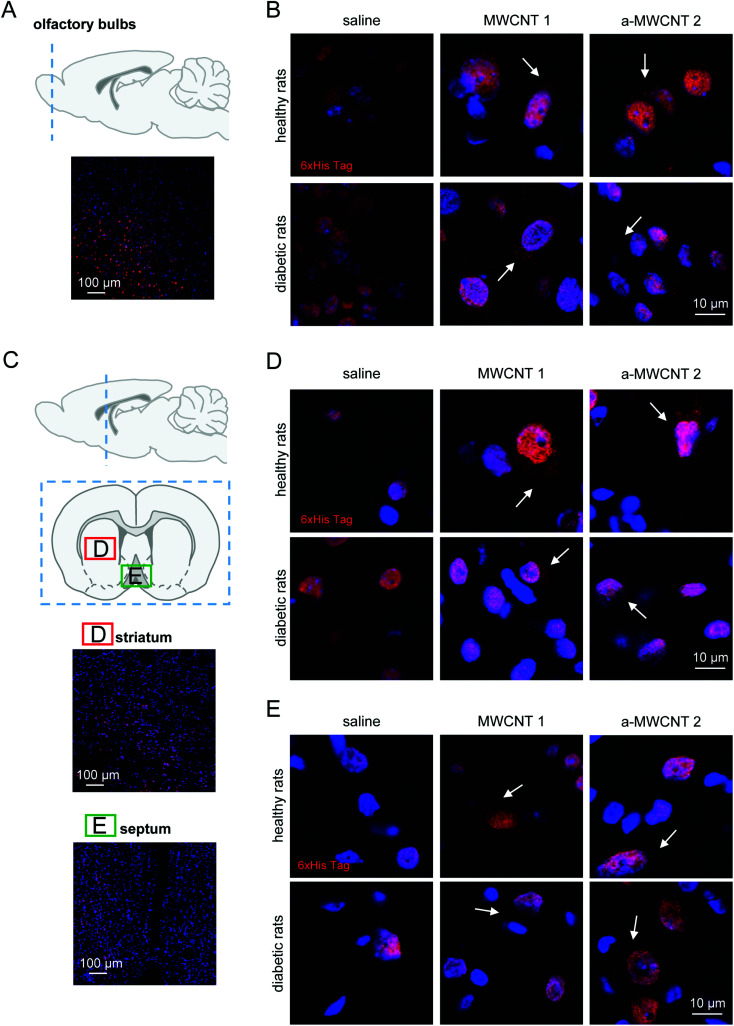
Tissue and cell localization of MWCNTs in olfactory bulbs, striatum and medial septum. (A and C) Schematic representation of the analyzed brain regions identified by dashed blue lines superimposed on a sagittal brain slice. Coronal brain slice (C) identifies the regions of striatum (D) and medial septum (E) analyzed and reported in representative low-magnification images. Immunostaining for His-tag (red) and nuclei (Hoechst; blue) in olfactory bulbs (B), striatum (D) and medial septum (E) of healthy (upper panels) and diabetic rats (lower panels) treated with saline, MWCNTs 1 and a-MWCNTs 2. White arrows indicate specific His-tag staining, which appears to be confined in the cytoplasm, while the nuclear labeling is to be considered non-specific, due to the presence of proteins (transcription factors) containing N-terminal residues of polyhistidine (see the main text for further details). *n* = 3 sections per rat, two/three animals in each experimental group were analyzed.

The olfactory bulbs (Fig. 2A) are the area more proximal to the olfactory epithelium and directly connected through the olfactory nerve axons to the CNT delivery site. Following the three-day administration protocol, the presence of the His-tag immunostaining was detected. Similar patterns of cell and tissue distribution of delivered CNTs have been detected in one-day-treated animals (data not shown). Notably, our preliminary analysis also revealed the early presence on CNTs in the piriform cortex (Fig. S8†), a brain area directly connected with the olfactory bulbs and projecting, among other brain regions, to the medio-dorsal thalamus.^39^

Non-specific nuclear staining was detected in saline-treated healthy and diabetic animals (Fig. 2B, left panels), probably due to the detection of poly-histidine tails in transcription factors prevalently located in cell nuclei.^40^ MWCNT delivery to healthy rats resulted in a detectable uptake of the nanomaterials into the cells, as demonstrated by the clear cytoplasmic immunostaining, not visible in the saline-treated group (Fig. 2B, upper panels, white arrows). Cellular uptake of MWCNTs was also evident in the olfactory bulb cells of diabetic rats (Fig. 2B, lower panels, white arrows). A general increase of the extracellular staining suggested a non-specific spread of the nanomaterials correlated with the occurrence of brain sufferance secondary to metabolic insult (Fig. 2B, lower panels, white arrows). The anti-His staining pattern in the olfactory bulbs of a-MWCNTs2a–MWCNTs 2 treated rats was slightly different from those observed after MWCNT 1 administration (Fig. 2B, lower panels). Indeed, the extracellular staining was less evident, while the intracellular localization of a-MWCNTs 2 resembled that one observed in the bulbs of healthy rats (Fig. 2B, upper panels). Thus, the amount of carbon nanomaterials reaching cells in the olfactory bulbs seemed to correlate with the type of CNTs used.

Our evaluation of CNT distribution was then carried out in a rostro-caudal plan analyzing striatum (Fig. 2C and D) and medial septum (Fig. 2C and E), two brain regions potentially affected by neurodegeneration following diabetes development.^27–29,41^ In healthy rats both MWCNTs were found mostly localized in the cytoplasm of striatal cells, while their presence in the extracellular milieu was not detected (Fig. 2D, upper panels, white arrows). The experimental diabetes affected the distribution of both CNTs in the striatal parenchyma, since MWCNT immune detection was evident also in the extracellular space (Fig. 2D, lower panels, white arrows). A consistent immunoreactivity for the His-tag was also detected in the medial septum (Fig. 2C and E), a nucleus belonging to the basal forebrain region. The presence of both MWCNTs was confined in the cell cytoplasm, while little or no presence at all was evident in the extracellular parenchyma, both in healthy and STZ-treated animals (Fig. 2E, white arrows). Moreover, any substantial differences in the features of MWCNT distribution in the medial septum were noticed when healthy and diabetic rats were compared. It is worth noting that the presented images are relative to a three-day protocol of delivery. The detection of MWCNTs in the striatum and medial septum, indeed, was very difficult in rats treated for one day (not shown), suggesting that the displacement of the CNTs in regions relatively far from the site of delivery requires longer administration time than a one-day protocol.

These data indicate that a three-day treatment protocol is sufficient to allow MWCNT localization in regions located in the rostral part of the brain and that the only parameter affecting their distribution in such regions is the presence of a putative neural degeneration, here induced by hyperglycemia.

**Fig. 3:**
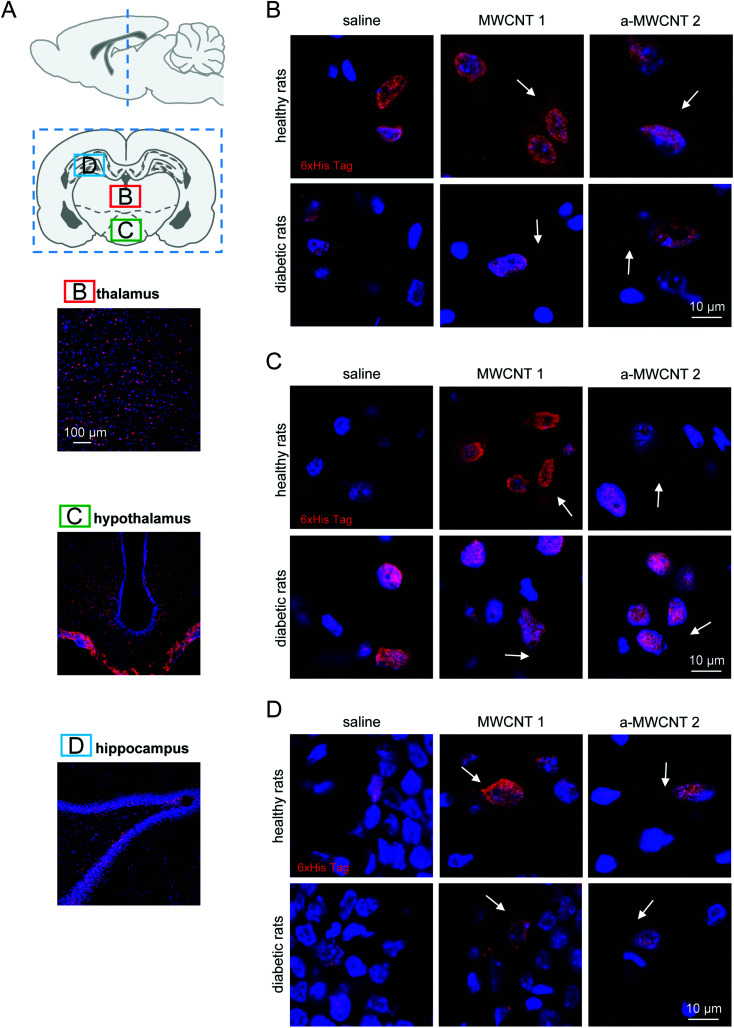
Tissue and cell localization of MWCNTs in the thalamus, hypothalamus and hippocampus. (A) Schematic representation of the analyzed brain regions identified by dashed blue lines superimposed on a sagittal brain slice. A coronal brain slice identifying the regions of thalamus, hypothalamus and hippocampus analyzed and reported in representative low-magnification images. Immunostaining for His-tag (red) and nuclei (Hoechst; blue) in thalamus (B), hypothalamus (C) and hippocampus (D) of healthy (upper panels) and diabetic rats (lower panels) treated with saline, MWCNTs 1 and a-MWCNTs 2. White arrows indicate specific His-tag staining. *n* = 3 sections per rat, two/three animals in each experimental group were analyzed.

The middle part of the brain was then analyzed (Fig. 3A). Both MWCNTs were easily and consistently detected in the thalamus (Fig. 3A and B), localized in the cytoplasm of thalamic cells in both healthy and diabetic rats, while they were never found in the extracellular space.

As observed in the olfactory bulbs, one day of CNT delivery was enough to detect the His-tag in thalamic cells (not shown). This evidence suggests that the thalamus is a preferential target for MWCNTs delivered through the olfactory route and that this kind of delivery is not affected by diabetic encephalopathy. The MWCNT pattern of distribution was similar in hypothalamic nuclei (Fig. 3A and C) in both healthy and diabetic rats but three-day administration was necessary to allow the immunostaining of the His-tag to be detected. Again, the His-tag was localized exclusively in the cell bodies. Apparently, though not quantified, the occurrence of diabetic-induced brain sufferance limited the distribution of both MWCNTs in the hypothalamus, since immunopositive cells were identified with increasing difficulty.

We then analyzed the potential presence of CNTs in the hippocampus after their intranasal delivery (Fig. 3A and D). No differences were found in the cellular distribution of the His-immunoreactivity when rats treated with MWCNTs 1 and a-MWCNTs 2 were compared. The MWCNTs were again found in the cell cytoplasm in both healthy and diabetic rats (Fig. 3D). It is worth noting that no His immunoreactivity was detected in the granular region of the dentate gyrus (DG), a region of dense-packed glutamatergic cells, neither in healthy nor in STZ-treated rats.

### 3.4. proNGF/mNGF ELISA

**Fig. 4:**
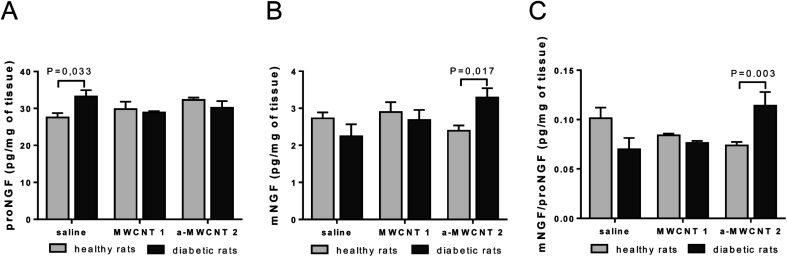
ELISA of mature NGF (mNGF) and proNGF hippocampal content. proNGF (A) and mNGF (B) levels measured as pg/total mg of tissue in healthy (clear columns) and diabetic rats (gray columns) intranasally delivered with saline, MWCNTs 1 and a-MWCNTs 2. (C) mNGF/proNGF ratio in hippocampus targeted by MWCNTs. Data are presented as mean ± SEM, *n* = 6–8, two-way ANOVA followed by the Bonferroni multiple comparison, *P* values reported in the figure.

We already demonstrated the ability of annealed MWCNTs to positively regulate the expression of molecular markers of neurodegenerative diseases, inducing the release of the neuroprotective factor mNGF *in vitro*.^12^ Following these findings, we aimed to verify the hypothesis that a-MWCNTs 2 was able to stimulate also *in vivo* a pro-neurotrophic function in the brain areas reached after their delivery *via* the olfactory route. Thus, we explored the potential of MWCNTs to affect the tissue balance between the neurotrophic mature NGF (mNGF) and its neurotoxic counterpart, the precursor proNGF^42^ (Fig. 4).

In the hippocampus, proNGF was affected neither by diabetes induction nor by CNT treatment, as revealed by the lack of significant effects for the two main factors in the ANOVA. However, a significant interaction between disease induction and CNT treatment was revealed [*F* (2, 34) = 4.260, *P* = 0.0223]. Bonferroni *post hoc* showed a significant increase of proNGF induced by STZ treatment (diabetic *vs.* healthy in the saline-treated rats, *P* value in Fig. 4A). As observed for proNGF, only a significant interaction between the two main factors was revealed by ANOVA for mNGF [*F* (2, 38) = 4.517, *P* = 0.0174]. The *post hoc* analysis showed that only the treatment with a-MWCNTs 2 was able to induce a significant increase of mNGF in the hippocampus of diabetic rats (diabetic *vs.* healthy a-MWCNTs 2-treated rats, *P* value in Fig. 4B). A significant interaction effect between the two main factors was only found for the ratio mNGF/proNGF in the ANOVA [*F* (2, 38) = 9.359, *P* = 0.0005]. The Bonferroni test confirmed that only the a-MWCNTs 2 treatment induced a significant increase in the mNGF/proNGF ratio (Fig. 4C), suggesting the triggering of neuro-protective effects by these CNTs.

Overall, these results indicate that electroconductive a-MWCNTs 2 could possibly stimulate a neuro-reparative mechanism in a diabetic brain, based on the stimulation of NGF neurotrophic activity.

### 3.5. Selective cellular uptake of CNTs by neurons and glia

**Fig. 5:**
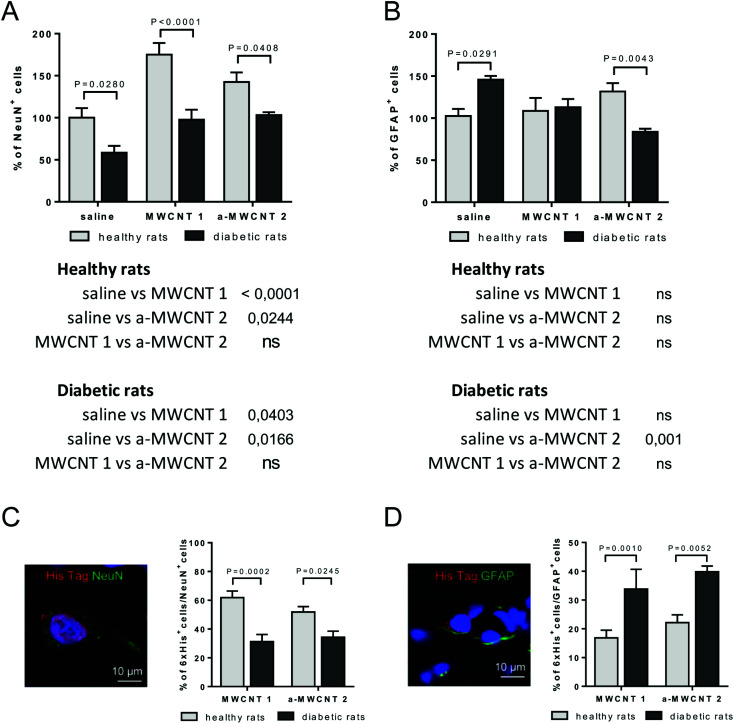
Effects of MWCNT treatment on NeuN^+^ and GFAP^+^ cells and on MWCNTs uptake by neurons and glia. NeuN^+^ (A) and GFAP^+^ cells (B) measured in the hilus of the hippocampus of healthy (clear columns) and diabetic rats (grey columns) treated with saline, MWCNTs 1 and a-MWCNTs 2. Data are presented as mean ± SEM and reported as percentage *versus* saline-treated healthy animals. *n* = 4–7, two-way ANOVA followed by Bonferroni multiple comparison. *P* values reported in the figure refer to pairwise comparisons between diabetic and healthy rats. The table below the graph shows *P* values of multiple comparisons among different intranasal treatments. (C) Representative picture of cells double-immunopositive for NeuN (neuronal marker) and His-tag (MWCNTs) with quantification of % NeuN^+^/His^+^ cells over the NeuN^+^ total cells. (D) Representative picture of cells double-immunopositive for GFAP (glial marker) and His-tag (MWCNTs) with quantification of % GFAP^+^/His^+^ cells over the GFAP^+^ total cells. *n* = 4–7, two-way ANOVA followed by Bonferroni multiple comparison. *P* values depicted in the figure.

We determined which types of brain cells were able to internalize MWCNTs delivered *via* the intranasal route. To achieve this goal, we performed several double and triple immunostaining, matching anti-His-tag antibodies with selective neuronal and glial markers. As a first approach, we analyzed the percentage of NeuN-positive or GFAP-positive DG-cells following intranasal delivery of saline, MWCNTs 1 or a-MWCNTs 2 (Fig. 5).

The percentage of NeuN^+^ cells was affected by both diabetes induction and MWCNT treatment, as revealed by the significant effects for the two main factors in the ANOVA (Fig. 5A, [*F* (2, 30) = 15.90, *P* < 0.0001], [*F* (1, 30) = 37.13, *P* < 0.0001]). Bonferroni *post hoc* showed a significant increase of NeuN^+^ cells induced by the treatment with the two MWCNTs in both healthy and diabetic animals (Fig. 5A tables below the graph). As expected, the percentage of NeuN^+^ cells significantly decreased in saline-treated diabetic rats (diabetic *vs.* healthy rats, *P* value shown in Fig. 5A). The percentage of NeuN^+^ cells was normalized in both MWCNTs 1- and a-MWCNTs 2-treated diabetic animals (diabetic *vs.* healthy rats, *P* values shown in Fig. 5A).

The percentage of GFAP^+^ cells (Fig. 5B) was affected neither by diabetes induction nor by MWCNT treatment, as revealed by the lack of significant effects for the two main factors in the ANOVA. However, a significant interaction between disease induction and MWCNTs treatment was revealed [*F* (2, 22) = 10.49, *P* = 0.0006]. Bonferroni *post hoc* did not show any variation of GFAP^+^ cells in healthy animals treated with both MWCNTs, while a significant decrease was observed in a-MWCNTs 2-treated diabetic animals (Fig. 5B tables below the graph). The diabetes induction significantly increased the number of GFAP^+^ cells in saline-treated rats (diabetic *vs.* healthy rats, *P* value shown in Fig. 5B). Of note, only the treatment with a-MWCNTs 2 normalized the percentage of GFAP^+^ cells in diabetic rats (diabetic *vs.* healthy rats, *P* value shown in Fig. 5B). These results may indicate that treatment with a-MWCNTs 2 could ameliorate the diabetes-induced reactive astrogliosis.

To analyze the percentage of NeuN^+^ and GFAP^+^ cells internalizing CNTs, co-localization experiments were performed in the brain of healthy and diabetic rats treated with MWCNTs 1 or a-MWCNTs 2 (Fig. 5C and D). The percentage of NeuN^+^/6xHis^+^ cells significantly decreased in both MWCNTs 1 and a-MWCNTs 2-treated diabetic animals (Fig. 5C). On the other hand, the percentage of GFAP^+^/6xHis^+^ cells significantly increased in both MWCNTs 1 and a-MWCNTs 2-treated diabetic animals (Fig. 5D). These results indicate that diabetes rendered glia more susceptible than neurons to internalize MWCNTs.

To discriminate which subtype of NeuN^+^ and GFAP^+^ cells is involved in the uptake of CNTs, we performed double immunofluorescence experiments focusing on cholinergic, catecholaminergic and GABAergic phenotypes and on microglia cells. Choline acetyl transferase (ChAT), tyrosine hydroxylase (TH), CD11b and Iba1 monoclonal antibodies were selected as specific markers of cholinergic cells (ChAT^+^), catecholaminergic cells (TH^+^), and microglia (CD11b^+^ or Iba1^+^), respectively. No MWCNT uptake was appreciable in any cell type (Fig. S9†).

**Fig. 6:**
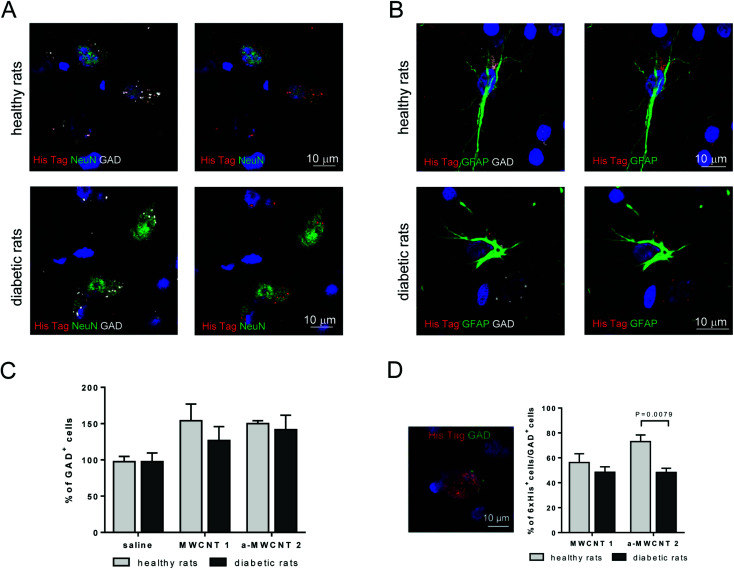
MWCNT displacement in GABAergic cells. Representative pictures of hilus cells immunostained for His-tag (red), GAD (grey) and, NeuN (green) (A) or His-tag (red), GAD (grey) and, GFAP (green) (B). (C) Percentage of GAD^+^ cells in healthy (clear columns) and diabetic rats (gray columns), treated with saline, MWCNTs 1, and a-MWCNTs 2. Data presented as percentage of saline-treated healthy rats. *n* = 4–7, two-way ANOVA. (D) Representative picture of double-immunopositive GAD^+^/His-tag^+^ cells and their percentage over the GAD^+^ total cells. *n* = 4–7, two-way ANOVA followed by Bonferroni multiple comparison. *P* values reported in the figure.

To characterize the possible selective uptake of MWCNTs by GABAergic cells, triple immunofluorescence experiments (NeuN/His-tag/GAD and GFAP/His-tag/GAD) were performed in the hilus of the hippocampus, the representative brain area targeted by the CNT delivery, of healthy and diabetic rats treated with a-MWCNTs 2 (Fig. 6).

His-tag immunoreactivity was found in the cytoplasm of GABAergic neurons (GAD^+^/NeuN^+^ cells) in both healthy and diabetic rats Fig. 6A. a-MWCNTs 2 were also found to internalize in GABAergic glial cells (GAD^+^/GFAP^+^ cells) in both healthy and diabetic rats (Fig. 6B). This result, indicating that almost exclusively GABAergic astrocytes and neurons are able to uptake MWCNTs *in vivo* after their intranasal delivery, corroborate the previously reported *in vitro* evidence of MWCNT internalization in mouse cortical GABAergic astrocytes.^43^ Moreover, although the percentage of total GAD^+^ cells did not vary neither after diabetes induction nor after treatment with both MWCNTs (Fig. 6C), the percentage of GAD^+^ cells that internalized a-MWCNTs 2 was significantly decreased in treated diabetic rats as compared to treated healthy rats (Fig. 4D).

### 3.6. Electrochemical properties of MWCNTs and their interaction with Ca^2+^, Zn^2+^ and Cu^2+^

Finally, because of the known role played by metal ions in the NGF and in the triggering of the NGF signal transduction,^44^ we aimed to verify whether the effect of a-MWCNTs 2 on the balance between the neurotrophic mNGF and its neurotoxic counterpart, proNGF, could be due to the sorption capacity on Cu^2+^ or Zn^2+^ cations. The electrochemical analysis of MWCNTs 1 and a-MWCNTs 2, as the film was deposited on ITO surface, was carried out in a PBS electrolyte solution (Fig. S2†). Cyclic voltammetry (CV) and open circuit potential (OCP) measurements were carried out (Fig. S3†).

The current–potential curves of the two types of MWCNTs, compared with the ITO substrate, showed a very similar pattern consisting of a pseudo-capacitive behavior, as already reported for the corresponding non functionalized materials, MWCNTs-P and a-MWCNTs-P.^11^ In particular, in agreement with the results of a previous study, a-MWCNTs 2 showed a higher electrical performance and capacitance with respect to MWCNTs 1 (Fig. S3†).

OCP studies were performed on MWCNT films, deposited on ITO, with increasing concentrations of three indications, namely Ca^2+^, Cu^2+^ and Zn^2+^, to ascertain any interaction between such ions and the CNTs (Fig. S2†). Indeed, such an interaction affects the OCP values depending on the type of ion and its concentration. It is possible to observe that Ca^2+^ ions produce a decrease of OCP by increasing their concentration only for a-MWCNTs 2, whereas for MWCNTs 1 the trend parallels that of ITO (substrate), taken as reference (Fig. S2A†). Such results are in agreement with those reported for similar MWCNTs in the paper by Serafino *et al.*^11^ It is also worth noting that while the presence of Zn^2+^ is ineffective on the OCP (Fig. S2C†), copper ions Cu^2+^ have a strong effect on the OCP of a-MWCNTs 2 (Fig. S2B†), very similar to the case of Ca^2+^ (Fig. S2A†). These results indicate that Cu^2+^ has a higher affinity towards a-MWCNTs 2 compared to Ca^2+^, as the fall of OCP is nearly complete within the 40 μM concentration of Cu^2+^, indicating a typical saturation behavior.

## 4 Discussion

Several research groups have investigated the biodistribution profile of CNTs using different administration routes and different tracking modalities to qualitatively and quantitatively determine the preferential site of accumulation of CNTs and the associated toxicity.^45^ Intravenously injected CNTs display the ability to reach almost every organ, including the brain, though in small quantities.^46–48^ Although CNT intravenous administration is of special value for the characterization of their tissue accumulation profiles, it seems to be disadvantageous since it leads to a widespread distribution in many organs.^47^ The interactions of MWCNTs with cortical brain tissue *in vivo* following stereotactic administration were investigated by Bardi *et al.*^20^ These authors reported that the different MWCNT chemical functionalization led to significant differences in nanotube localization and distribution patterns within the brain parenchyma, along with distinct differences in cellular uptake and inflammatory reactivity. Indeed, any application that involves implantation or injection of material directly into the brain needs to address the question of inflammatory responses triggered following the interaction with the CNS tissue. The possibility to deliver drugs to the brain parenchyma by a less invasive technique has been reported, exploiting the olfactory route by exposure of the nasal epithelium to a suitable concentration of the desired therapeutic substance.^49–53^ Intranasal delivery is a non-invasive method that rapidly targets therapeutics to the CNS, bypassing the BBB and minimizing systemic exposure. Through intranasal administration therapeutics can rapidly gain access to the CNS along olfactory nerve pathways, leading directly to the CNS from the nasal cavity.^54^ The possibility of delivering CNTs to the brain *via* the olfactory route has been recently put forward,^55–57^ but their biodistribution in the brain after intranasal administration remains an open question.

Our current study aimed at filling this specific gap of knowledge. Nowadays, there is a serious concern about how to treat neurodegenerative diseases, and strategies aimed at stimulating an endogenous, NGF-based, healing process are warranted in diseases like Alzheimer's or traumatic brain injuries.^58^ We assessed the brain tissue and cellular distribution of two types of MWCNTs. To minimize the interference of the tissue fluorescence with the nanotube reporting systems, we decided to decorate their surface using a hexa-histidine tag, which can be specifically recognized by suitable antibodies. The use of the His-tag on nanotubes was previously reported using the hexa-histidine derivative linked to a pyrene moiety able to interact with the CNT surface by supramolecular interactions.^59^ Herein we reported the covalent tube functionalization with the His-tag, in two steps. First the Boc-protected benzylamine was introduced linked to the MWCNTs by the Tour reaction, then, after deprotection of the amine, the His-tag was allowed to react to form the corresponding amide and another deprotection of the Fmoc group is necessary to obtain a-MWCNTs 2. The arylation of the CNTs is a well-known and widely used approach to efficiently and conveniently modify the nanotubes.^60,61^ Both in the case of MWCNT-P and a-MWCNT-P, the functionalization reaction resulted to be efficient with a degree of functionalization in the same order of magnitude for the two materials (around 500 μmol g^−1^) as well as the following coupling, resulting in a degree of functionalization with the His-tag of around 50 μmol g^−1^. The functionalization of MWCNTs 1 and a-MWNCT2 being comparable, the differences in the biological characteristics cannot be attributed to the decoration, which results in being consistent in the two samples. Moreover, the introduced surface modification did not alter the electroconductive characteristics of the two constructs, considering that those are conserved with respect to the non functionalized nanomaterials. In fact, as previously shown, a-MWCNTs possess electroconductive characteristics, already proven to be effective in modulating microglia activation *in vitro*, inducing a phenotype switch toward the anti-inflammatory M2-microglia while the other one (MWCNTs) does not present the same electroconductive behavior.^12^

Since data on the brain tissue diffusive capabilities after intranasal delivery of CNTs are completely lacking, as a first approach we performed one and three-day administrations of these materials by intranasal delivery, with the aim of obtaining indications about the possible mechanisms of CNT diffusion into the brain parenchyma. The displacement of therapeutic molecules in the brain is generally affected by the physio-pathological condition of the animal. Thus, in order to explore the hypothesis that CNTs could affect the tissue balance between the neurotrophic mature NGF (mNGF) and its neurotoxic counterpart, the precursor proNGF,^42^ we delivered MWCNTs to both healthy and diabetic rats, the latter known to be affected by mild cognitive impairment associated with neurodegeneration and dysregulation of NGF production and activity, especially at the hippocampal level.^27–29^

A single administration resulted in the detection of both MWCNTs in the olfactory bulbs, the piriform cortex and thalamus, while the three-day treatment regimen allowed both MWCNTs to reach structures in the basal forebrain, striatum, hypothalamus, thalamus, hippocampus and cortex. Our findings about the prevalent or sole distribution of MWCNTs to brain regions involved in olfaction^39^ after a single administration indicate that the olfactory nerve pathway and the intraneural diffusion from the bulbs to thalamus through the piriform cortex may be involved earlier.^62^ On the other hand, the presence of immune reactive nanomaterial in other brain regions, exclusively after 3 days of intranasal treatment, suggests that intraneural and vascular/lymphatic/extracellular mechanisms of diffusion into the parenchyma may be involved later.

We found that only GABAergic cells were capable of internalizing MWCNTs 1 and a-MWCNTs 2, in healthy and diabetic rat brains. Notably, both samples were found in the cell cytoplasm of the Dentate Gyrus (DG) of the hippocampus in healthy and diabetic rats, but no His immunoreactivity was detected in the granular region of DG, a region of dense-packed glutamatergic cells, neither in healthy nor in STZ-treated rats. Dentate Gyrus is a hippocampal region corresponding to the first relay station in the classical tri-synaptic loop circuit that elaborates information coming from the entorhinal cortex. Notably, this is also one of the two brain areas in which an active adult neurogenesis process has been demonstrated.^63^ Due to its selective and high vulnerability to the metabolic stress induced by hyperglycaemia^27–29^ and its involvement in the development of major cognitive diseases,^63^ the hippocampus represents a privileged brain area to be targeted by neuroprotective therapies. The demonstration of a close correlation between the potential neuroprotective effect of MWCNTs and neurodegeneration, is lacking in the present study. Nevertheless, our data represent the first evidence that MWCNTs may be ascribed to the category of experimental neuroprotective agents, possibly useful as a promoter of NGF-based neuro reparative therapies.^58^

Though the specific supporting literature is lacking, a previous study indicated that mouse cortical GABAergic astrocytes were selectively internalizing MWCNTs *in vitro*.^43^ We extended this observation demonstrating that, not only GABAergic astrocytes, but also GABAergic neurons selectively uptake MWCNTs *in vivo*. It is difficult to speculate about the mechanism underlying such a selective uptake. Further experiments are underway *in vivo* to unravel the reasons why MWCNTs are internalized exclusively by GABAergic cells and to investigate their effects on the functioning of these cells.

The tissue and cell distribution of both types of MWCNTs in the brain of healthy and diabetic rats did not present qualitative macroscopic variances. However, significant differences were found when the effects of the treatments were evaluated on mNGF/proNGF protein levels and the glial/neuronal phenotypes in the hippocampus. In light of previous reports, demonstrating the relevance of correcting the hippocampal proNGF/mNGF unbalance induced by diabetes to achieve neuroprotection in such animal models,^28,29,36^ our data on the mNGF and proNGF protein modulation after CNT delivery indicate that such treatment could stimulate neuroprotection after diabetes induction. Though limited to a single specific neuronal/glial phenotype, the intranasally delivered MWCNTs resulted in effects on a molecular system (NGF), mainly produced and modulated in GABAergic cells.^64–66^ A reasonable working hypothesis to explain this effect should first rely on the electro-conductive specificity of annealed MWCNTs. We previously demonstrated that the a-MWCNT-P sample used for the functionalization in the present study, which were purified by annealing of MWCNTs-P, affected Ca^2+^ ions balancing between extra- and intracellular compartments in electrically sensitive rat cells (pituitary corticotropin-derived cell line). This effect was due to the sorption of Ca^2+^ cations on the CNT surface because of the excess of electrons on the aromatic units formed on MWCNTs after annealing.^11^ Many pieces of evidence suggest that metal ions could play a role in the NGF and NGF signal transduction triggering.^44^ Cu^2+^ ions have been shown to play a critical role in the modulation of the biological activity of NGF^67^ and to possess anti-inflammatory effects on microglia and astrocytes *in vitro*. In order to verify if, as occurred with Ca^2+^, there was sorption of Cu^2+^ cations to a-MWCNTs 2, we evaluated the changes in the electronic properties of the two materials upon the addition of Cu^2+^ to the PBS solution, by Open Circuit Potential (OCP) measurements. The addition of Cu^2+^ modified significantly the OCP value only of a-MWCNTs 2 films, thus evidencing their strong interaction with copper cations. It is likely that the interaction between Cu^2+^ cations and electro-conductive a-MWCNTs 2 could affect the metal homeostatic machinery involved in the NGF signalling, thus promoting changes in the NGF phenotype and function towards neuroprotective effects.

## 5 Conclusions

The exploration of delivering CNTs to the CNS in an efficient and non-invasive way was specifically driven by our previous observation concerning the ability of electroconductive MWCNTs to change the phenotype and function of microglial cells, positively regulating the expression of neuroprotective molecular markers such as mature NGF.^12^ Herein, we revealed that both MWCNTs delivered through the olfactory epithelium consistently diffused into the brain parenchyma, reaching at least medial regions of the brain, following the rostro-caudal axis, such as the hippocampus, thalamus and hypothalamus. The presence of CNTs in specific brain regions is associated with outcomes at molecular and cellular levels, which could be part of a CNT-induced neuroprotective response in a brain area having a particular relevance in the development and progression of certain neurodegenerative diseases.^68,69^ It is worth mentioning, however, that this preliminary study, besides demonstrating the possibility to deliver CNTs to the brain by intranasal administration, suggests that such treatment stimulated molecular outcomes. Indeed, after this preliminary report, a more complete and exhaustive pharmacology study which, among other things, quantifies the spread of CNTs in different regions of the brain and more extensively analyses pharmacokinetic and pharmacodynamic parameters and potential toxicity is deserved.

In all the regions reached by the nanomaterials, both MWCNT samples were internalized by neurons and glia expressing the GABAergic marker GAD. Only the treatment with a-MWCNTs 2 was able to induce a significant increase of mNGF in the hippocampus of diabetic rats, suggesting that they could stimulate a neuroreparative mechanism in a diabetic brain, based on the stimulation of NGF neurotrophic activity.

These two elements make CNTs potentially suitable to deliver therapeutics to cells and areas deeply involved in the development and progression of major neurological diseases with a strong GABAergic involvement, such as psychotic diseases^70,71^ or movement disorders.^72,73^ Our study also indicates that a three-day administration protocol is sufficient to achieve a consistent diffusion of MWCNTs to almost every analyzed brain region in the used animal models. However, further studies are ongoing on the clearance, toxicity and bioavailability of these materials, administered in different dosages in order to gain a more complete picture of the basic pharmacokinetics and the safety relative to MWCNT delivery to the brain.

To the best of our knowledge, this is the first study showing that MWCNTs delivered *via* the intranasal route are able to reach numerous brain areas and in particular the limbic area that plays a crucial role in the development and progression of major neurodegenerative diseases. Furthermore, most important from a translational standpoint, electroconductive a-MWCNTs 2 were demonstrated to be potentially able to exert a neuroprotective effect through the modulation of a key neurotrophic factor.

## Conflicts of interest

There are no conflicts to declare.

## Supplementary Material

NA-003-D0NA00869A-s001
