# Supplementary material for: Distribution in the brain and possible neuroprotective effects of intranasally delivered multi-walled carbon nanotubes
Source: Nanoscale Adv. 2020 Nov 12;3(2):418–31. doi: 10.1039/d0na00869a (PMC9418508; doi:10.1039/d0na00869a)
Supplement: NA-003-D0NA00869A-s001 [file NA-003-D0NA00869A-s001.pdf]

Electronic Supplementary Material (ESI) for Nanoscale Advances.
This journal is © The Royal Society of Chemistry 2020

Distribution in the brain and possible neuroprotective effects of intranasally delivered Multi-Walled Carbon Nanotubes

M. Soligo[a], F. M. Felsani[a], T. Da Ros[b], S. Bosi[b], E. Pellizzoni[b], S. Bruni[c], J. Isopi[d], M. Marcaccio[d], L. Manni[a], S. Fiorito[a*]

a. Institute of Translational Pharmacology - CNR, via Fosso del Cavaliere 100, 00133 Rome, Italy
b. INSTM – Trieste Unit & Department of Chemical and Pharmaceutical Sciences, University of Trieste, Via Licio Giorgieri 1, 34127 Trieste, Italy.
c. Former Medical Director Sanofi - Genzyme, Italy; currently Orchard Therapeutics, 108 Cannon Street, London, UK.
d. Department of Chemistry "G. Ciamician", University of Bologna, via Selmi, 2, 40126 Bologna, Italy

*Corresponding author e-mail: silvana.fiorito@ift.cnr.it

SUPPLEMENTARY MATERIAL

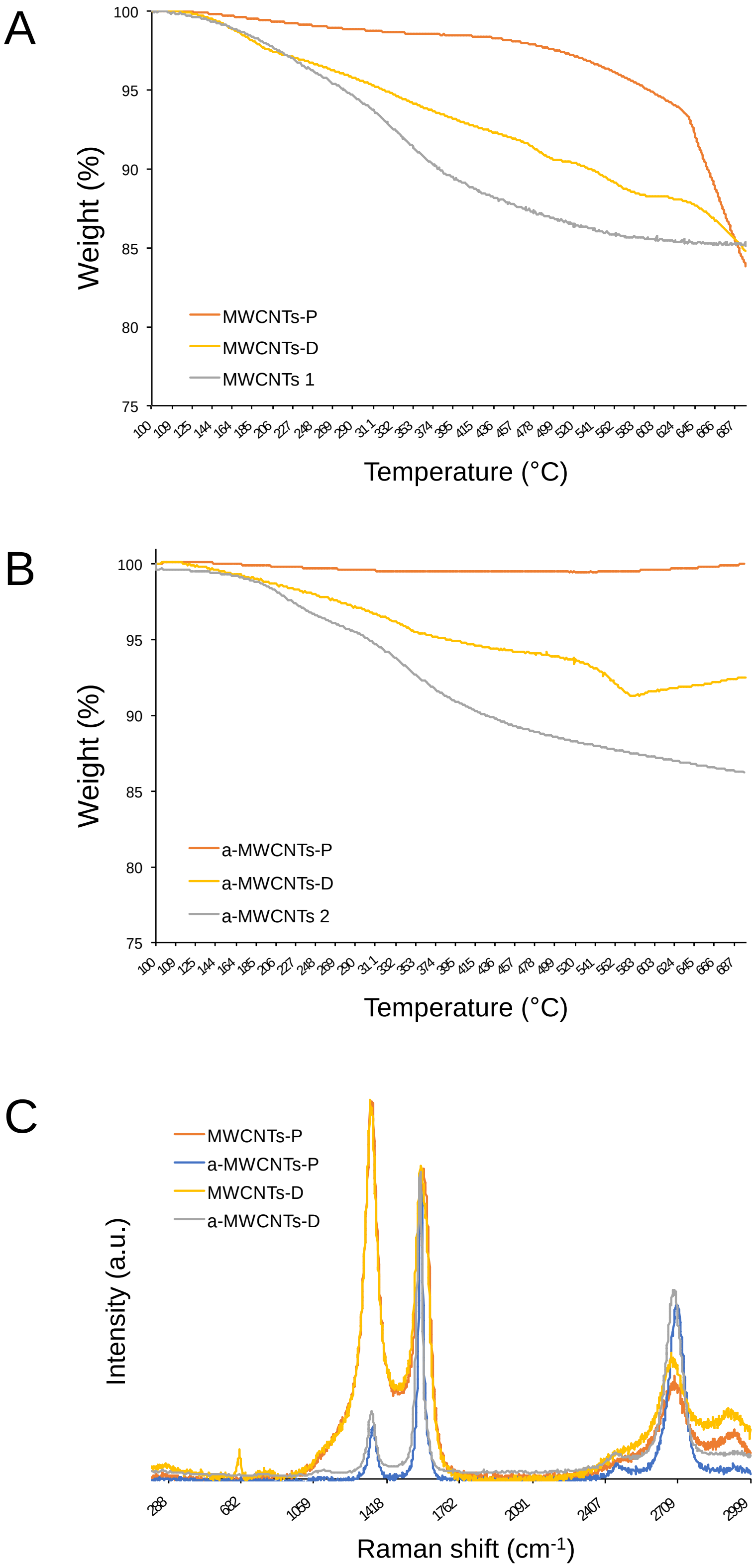

Figure S1. Characterization of MWCNTs 1 and a-MWCNTs 2.

(A) TGA analyses of the pristine, deprotected and His-tag conjugated MWCNTs; (MWCNTs-P, MWCNTs-D, and MWCNTs 1 respectively) (B) TGA analyses of the pristine, deprotected and His-tag conjugated annealed MWCNTs (a-MWCNTs-P, a-MWCNTs-D, and a-MWCNTs 2 respectively); (C) Raman spectra of pristine and deprotected derivatives.

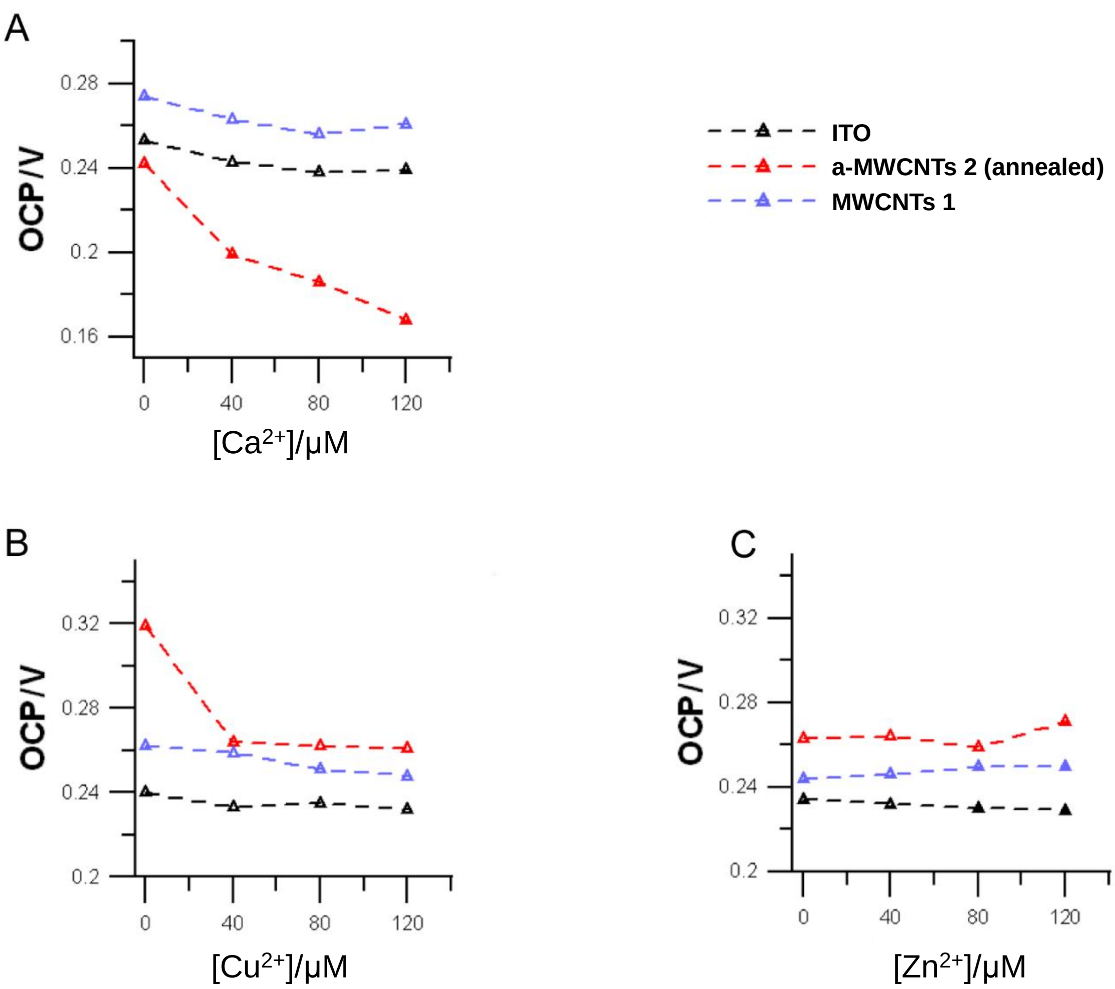

Figure S2. Electrochemical results and interaction of CNTs with Ca^{2+}, Cu^{2+} and Zn^{2+}.

Open circuit potentials of samples ITO (Black symbols and lines), a-MWCNTs 2 (red symbols and lines) and MWCNTs 1 (electric-blue symbols and lines) at various concentrations of (A) Ca^{2+}, (B) Cu^{2+} and (C) Zn^{2+}. The MWCNT samples were drop-casted on ITO (forming a 6 mm diameter disk) with a loading of 40 µl of MWCNT dispersion in PBS. Electrolyte solution: PBS 1x; reference electrode: SCE.

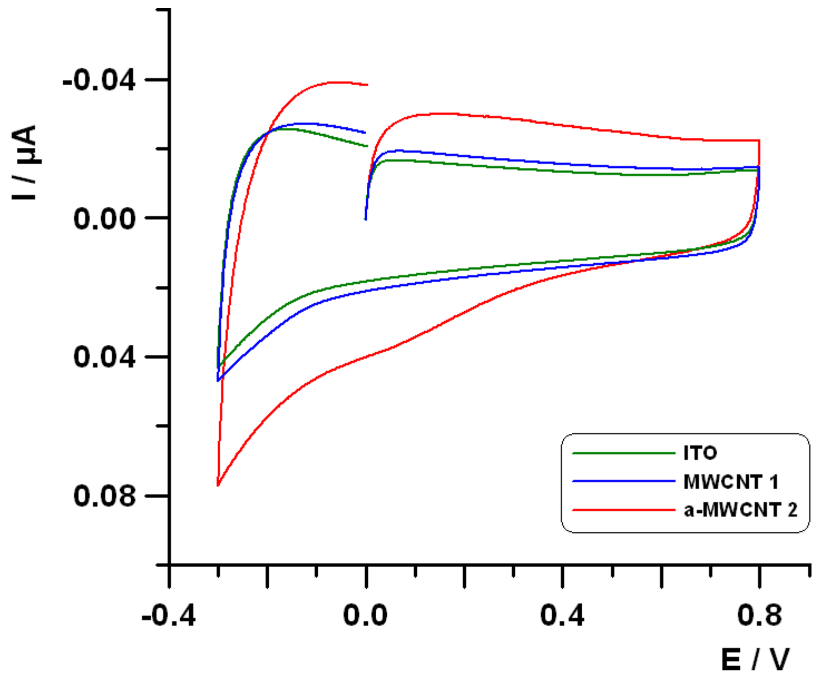

Figure S3. Cyclic voltammetric curves of CNTs.

Cyclic voltammetric curves of ITO (green line), MWCNTs 1 (drop-casted on ITO substrate - blue line) and MWCNTs 2 (drop-casted on ITO surface - red line) in a PBS 1x electrolyte solution at a scan rate of 0.1 V/s; active electrode surface: 0.22 cm2; reference electrode: SCE

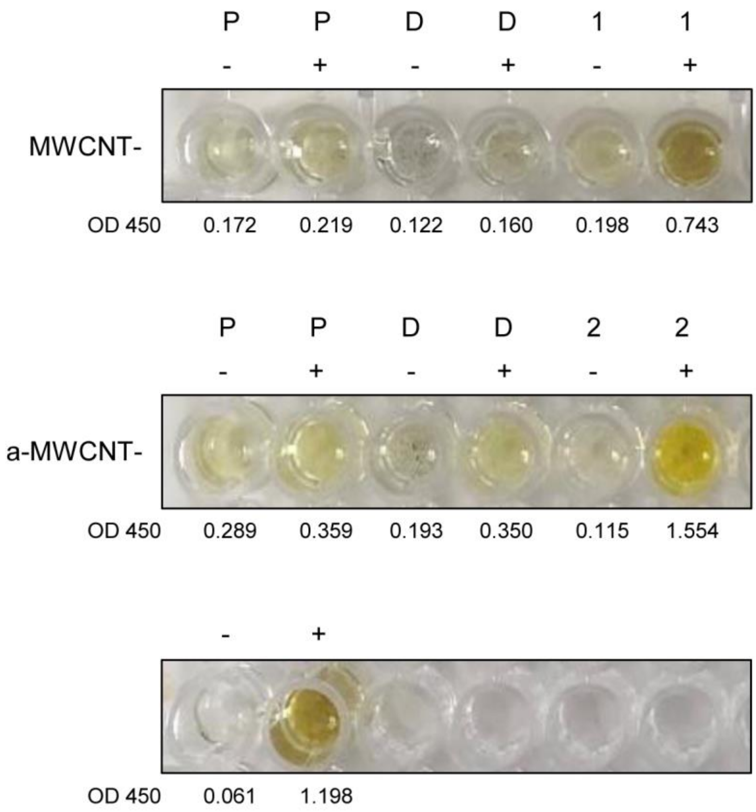

Figure S4. Assessment of MWCNTs functionalization.

Dispersed MWCNTs (first lane) and a-MWCNTs (second lane) were incubated with mouse anti-6xHis antibody and with anti-mouse-HRP antibody (+ conditions only). To visualize antibody reactivity, the chromogenic substrate 3',3',5',5'-tetramethylbenzidine (TMB) was used. The development of the colorimetric reaction indicates the presence of the 6xHis tag associated with the MWCNTs and was measured in absorbance mode at 450 nm.

Abbreviations: P = MWCNT-P and a-MWCNT-P respectively; D = MWCNT-D and a-MWCNT-D respectively; 1 = MWCNTs 1; 2 = a-MWCNTs 2; - = primary antibody only; + = primary plus HRP-secondary antibody.

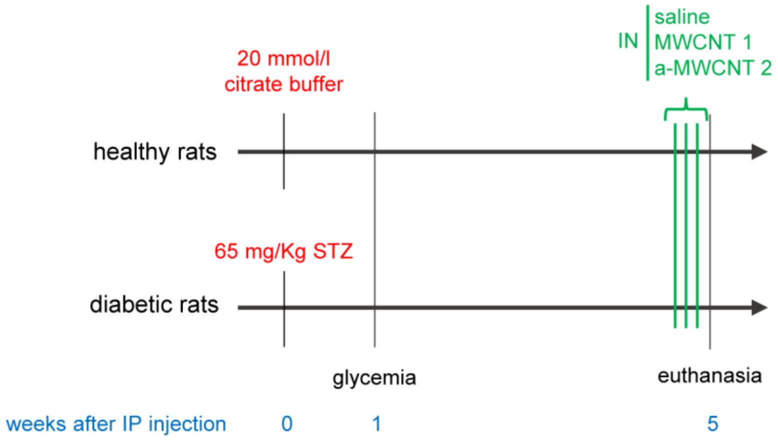

Figure S5. Rats experimental design.

Diabetes was induced by a single intraperitoneal injection of 65 mg/kg streptozotocin (STZ) dissolved in citrate buffer, pH 4.5 One week after STZ treatment, rats were checked for the establishment of diabetes. Before euthanasia, healthy and diabetic conscious rats were exposed to intranasal (IN) administrations of MWCNTs 1 or a-MWCTNs 2 or saline once a day for three days or for one day.

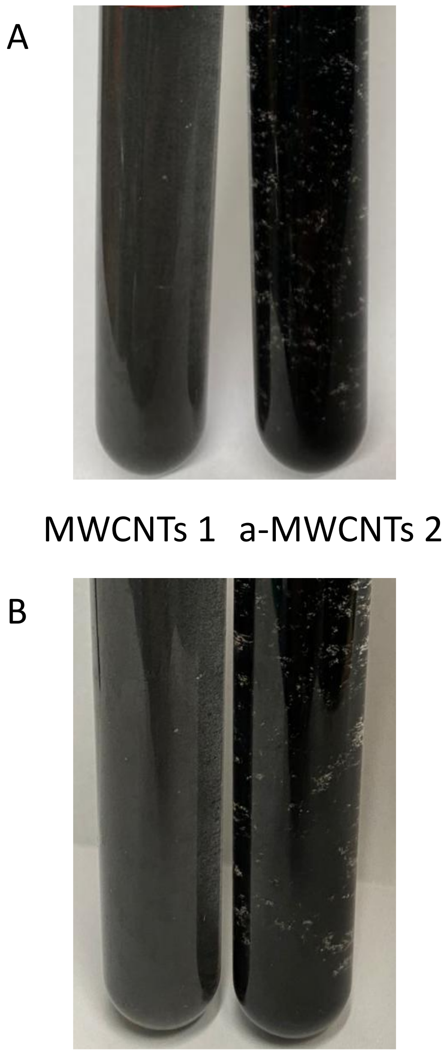

Figure S6. Dispersion of CNTs.

Suspension of MWCNTs 1 (left) and a-MWCNTs 2 (right) after 1 h of sonication (A) and after 1 h of sedimentation and followed by manual agitation (B). Preparation in sterile saline at 2 mg/mL concentration.

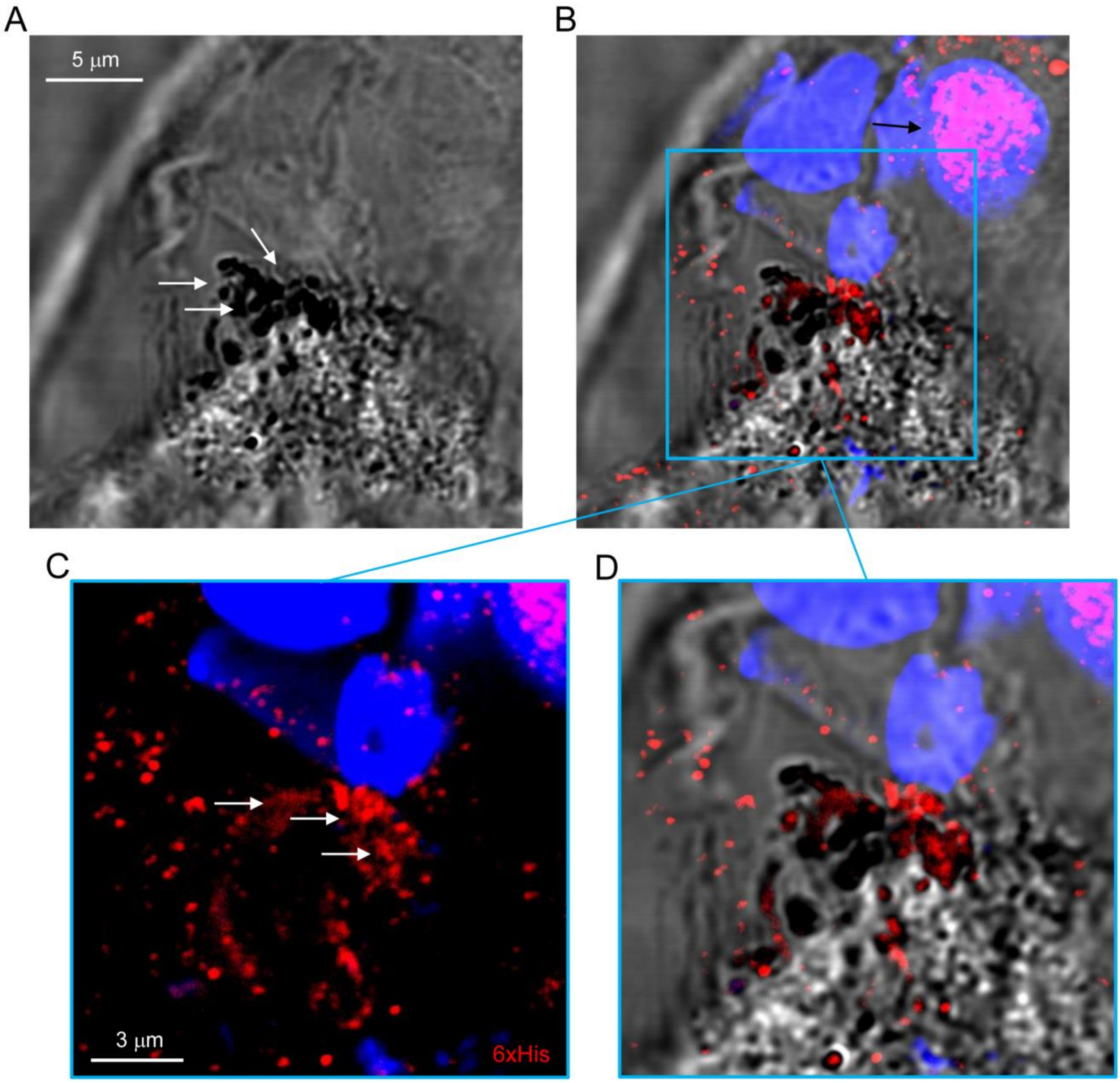

Figure S7. Intracellular localization of MWCNTs in Thalamus after intranasal delivery.

(A) Bright field image of selected area. Objective 63X, zoom 5. CNTs are detected as black-coloured particles (white arrows) (B) Bright field and confocal image were merged. Blue staining: Hoechst (nuclear). Red staining: His-tag. The presence of nuclear non-specific staining (black arrow) is conceivably due to the interaction of anti-His antibody with nuclear proteins (transcription factors mainly) bearing poly-His tail (see main text for details). (C) Confocal image magnification of the blue boxed area in panel (B). Most of the red-stained His-immunoreactivity is detected in cell cytoplasm (white arrow). (D) Merge of bright field and confocal image corresponding to the blue boxed area in panel (B). His-staining (red) and CNTs (black particles/aggregates) are mostly colocalized.

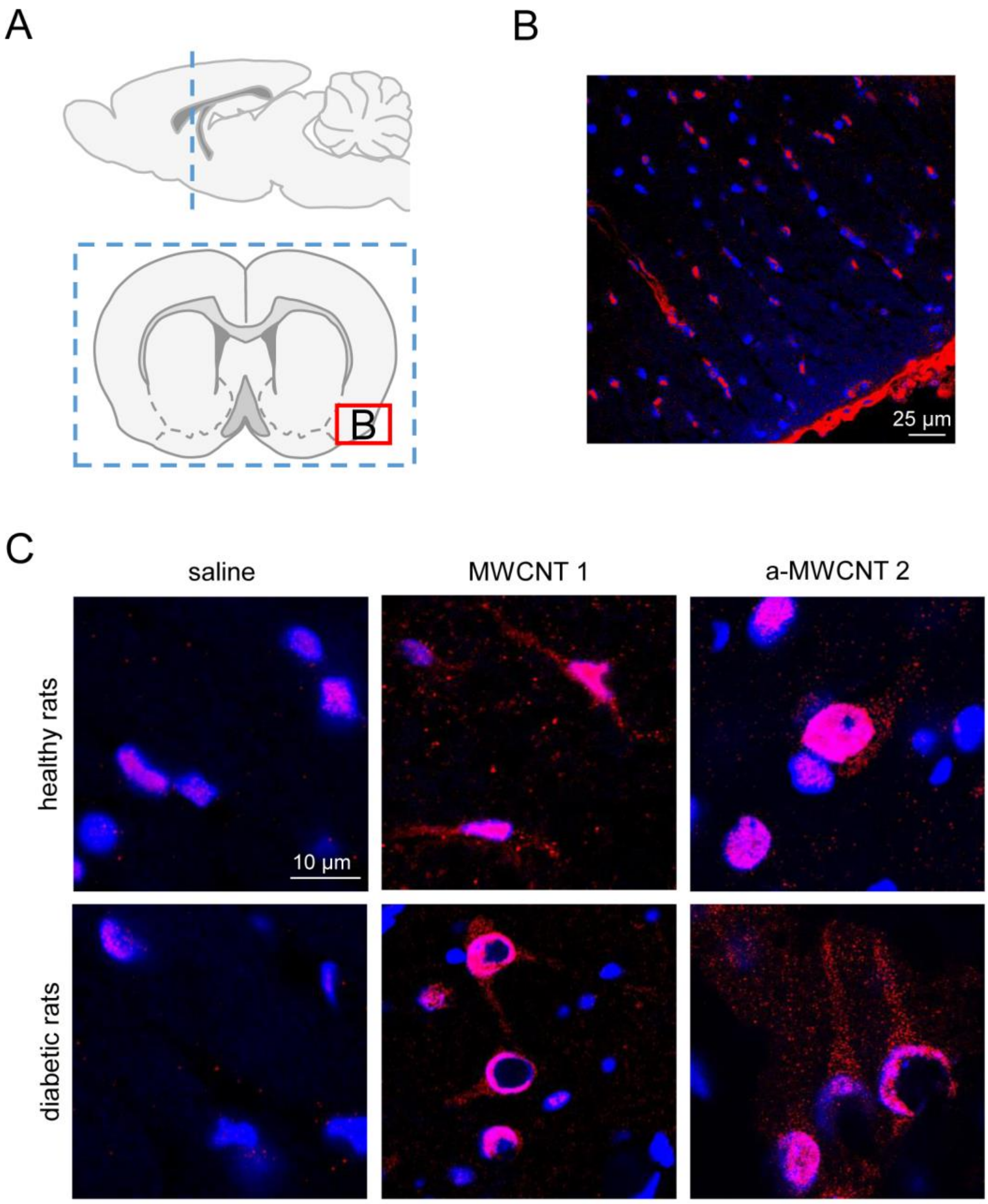

Figure S8. Tissue and cell localization of MWCNTs in Piriform Cortex.

(A) Schematic representation of the analyzed brain regions identified by dashed blue lines superimposed on sagittal brain slice. Coronal brain slice identifies the regions of Piriform Cortex analyzed and reported in representative low-magnification images (B). (C) Immunostaining for His-tag (red) and nuclei (Hoechst; blue) in Piriform Cortex of healthy (upper panels) and diabetic rats (lower panels) treated with saline, MWCNTs 1 and a-MWCNTs 2. n=3 sections/rat, two/three animals in each experimental group were analyzed.

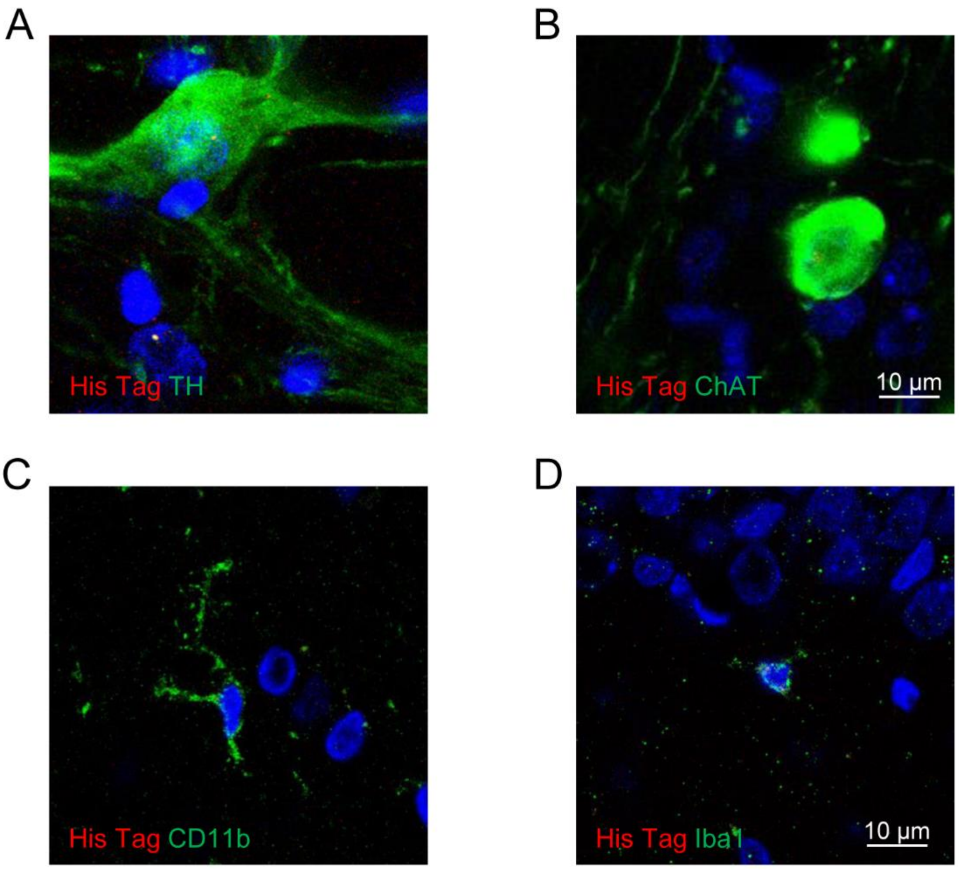

Figure S9. MWCNTs uptake in different cell types.

Double immunostaining for tyrosine hydroxylase (TH)/His-tag (A), choline-acetyl transferase (ChAT)/His-tag (B), CD11b/His-tag (C) and Iba1/His-tag (D) in selected brain areas. We failed to identify MWCNTs uptake in any cell identified for specific marker of cholinergic, catecholaminergic, and microglia.

Table S1. List of used antibodies.

Primary antibody (catalog, manufacture)	*Application/ Dilution*	*Secondary antibody (catalog, manufacture, dilution)*
mouse **anti-6xHis** (ab18184, Abcam)	IF: 1:500	**Donkey anti-mouse Alexa-Fluor 488** A32766 Invitrogen 1:200
rabbit anti-**His Tag** (12698, Cell Signaling)	IF: 1:400	**Donkey anti-rabbit Alexa-Fluor 555** A31572 Invitrogen 1:200
mouse **anti-NeuN** (90228, Immun. Sciences)	IF: 1:400	**Donkey anti-mouse Alexa-Fluor 488** A32766 Invitrogen 1:200
mouse **anti-ChAT** (gift of dr. Cozzari C.)	IF: 1:400	**Donkey anti-mouse Alexa-Fluor 488** A32766 Invitrogen 1:200
goat **anti-GAD** (7513, Santa Cruz)	IF: 1:50	**Donkey anti-goat Alexa-Fluor 647** A-21447 Invitrogen 1:200
mouse **anti-TH** (10593, Immun. Sciences)	IF: 1:400	**Donkey anti-mouse Alexa-Fluor 488** A32766 Invitrogen 1:200
mouse **anti-IBA1** (MA5-27726, Invitrogen)	IF: 1:50	**Donkey anti-mouse Alexa-Fluor 488** A32766 Invitrogen 1:200
mouse **anti-GFAP** (556327, BD Biosciences)	IF: 1:400	**Donkey anti-mouse Alexa-Fluor 488** A32766 Invitrogen 1:200
rabbit **anti-CD206** (ab64693, Abcam)	IF: 1:100	**Donkey anti-rabbit Alexa-Fluor 555** A31572 Invitrogen 1:200
mouse **anti-CD11** (ab8879, Abcam)	IF: 1:100	**Donkey anti-mouse Alexa-Fluor 488** A32766 Invitrogen 1:200
rabbit **anti-proNGF** (EP1318Y, Abcam 68151)	Detection ELISA: 1:5000	**HRP-linked anti-rabbit IgG** 7074, Cell Signaling 1:1000
goat **anti-NGF** (AF-556-NA, R&D)	Capture ELISA: 0.4 µg/ml	
mouse **anti-NGF 27/21** (52602, Millipore)	Detection ELISA: 1:1000	**HRP-linked anti-mouse IgG** 7076, Cell Signaling 1:1000

IF: Immunofluorescence
